# Case Report: Successful treatment of *Bartonella henselae*-associated crescentic glomerulonephritis in renal transplant recipient

**DOI:** 10.3389/fmed.2026.1854618

**Published:** 2026-05-28

**Authors:** Sophie Pierson, Melchior Chabannes, Stéphane Lang, Mathilde Colladant, Jean Seibel, Jamal Bamoulid, Didier Ducloux, Cécile Courivaud, Sophie Felix, Thomas Crepin

**Affiliations:** 1 Department of Nephrology, Dialysis, and Renal Transplantation, University Hospital of Besançon, Besançon, France; 2 Université de Franche-Comté, CHU Besançon, EFS, INSERM, UMR RIGHT, Besançon, France; 3 Department of Pathology, University Hospital of Besançon, Besançon, France

**Keywords:** *Bartonella henselae*, case report, crescentic glomerulonephritis, infectious glomerulonephritis, renal transplantation

## Abstract

**Background:**

Infectious crescentic glomerulonephritis (CrGN) is a rare but severe complication in kidney transplant (KTx) recipients. *Bartonella henselae*, typically associated with cat-scratch disease or infective endocarditis has rarely been reported as a cause of isolated glomerulonephritis in renal transplant recipients without infective endocarditis.

**Case presentation:**

A 64-year-old man with a history of two kidney transplants presented with a 3-week deterioration in renal function, progressing to nephritic syndrome (urine protein creatinine ratio (UPCR) 5.1 g/g, albumin 22 g/L, hypertension, and peripheral edema [+6 kg over 1 week]) and oliguric acute kidney injury (peak creatinine 684 μmol/L). Renal biopsy showed CrGN with IgM/IgA/C3 deposits, suggesting immune complex-mediated pathology. Initial investigations, including blood cultures, two transthoracic echocardiograms performed 1 week apart, and PET scan, ruled out infective endocarditis. Reinterrogation post-biopsy uncovered a cat bite 2 months prior, raising concerns for *Bartonella henselae* infection, which was confirmed by serological testing.

**Management and outcome:**

The patient received azithromycin (500 mg/day for 5 days) for infection and corticosteroids (5 mg/kg pulse followed by a tapering regimen) for glomerulonephritis. After 2 dialysis sessions, renal function improved as evidenced by recovery urine output, which led to discontinuous of dialysis. A major complication (upper gastrointestinal bleeding) required endoscopic intervention and blood transfusions. Nephroprotective therapy was optimized (irbesartan up titrated from 75 to 150 mg/days and dapagliflozin 10 mg/day introduced), leading to normalization of proteinuria (UPCR 0.5 g/g) at 1-year follow-up.

**Conclusion:**

This case underscores the importance of reinterrogating patients for exposure risks (e.g., animal bites) when facing unexplained CrGN, particularly in renal transplant recipients. It also highlights the effectiveness of combined antibiotic and corticosteroids therapies in managing infectious glomerulonephritis. Clinicians should consider *Bartonella* as a potential etiology in renal transplant patients with CrGN, even in the absence of classic symptoms.

## Introduction

*Bartonella henselae*, a gram-negative bacillus transmitted primarily through cat scratches or bites, is the causative agent of cat-scratch disease (CSD) described in 1950 by Debré (1). The frequency of Bartonella in cats, which is generally asymptomatic, exceeds 50% in endemic regions (2). While CSD typically presents as self-limited regional lymphadenopathy in immunocompetent individuals, immunocompromised patients—particularly solid organ transplant recipients—are at risk of disseminated and life-threatening complications (2–4). In fact, in solid organ transplant recipients, *Bartonella* infections pose significant diagnostic challenges due to their non-specific clinical presentations. Fever, granulomatous lesions, and angioproliferative disorders may mimic post-transplant lymphoproliferative disease including hepatic granulomatosis, bacillary angiomatosis, and, less commonly, infective endocarditis (5–7).

Renal involvement in *Bartonella* infections is rare but increasingly recognized, often manifesting as post-infectious immune complex-mediated glomerulonephritis. Histological patterns include mesangial, endocapillary, or extracapillary proliferation, as well as membranoproliferative glomerulonephritis (8, 9). These renal lesions are frequently associated with concurrent culture-negative infective endocarditis, which complicates up to 9% of *Bartonella* infections (10). However, renal manifestations in the absence of endocarditis remain poorly documented, particularly in kidney transplant (KTx) recipients (11–13).

Here, we report a new rare case of crescentic glomerulonephritis (CrGN) associated with *Bartonella henselae* infection in a KTx recipient, occurring in the absence of infective endocarditis. This case highlights the importance of considering *Bartonella* as a potential etiology in KTx recipients presenting with post infectious glomerulonephritis, even in the absence of classic CSD manifestations. We discuss the diagnostic approach, therapeutic challenges, and long-term implications for graft function.

## Case presentation

A 64-year-old man with a history two kidney transplants, performed 25 and 14 years prior for Goodpasture disease, was admitted to the nephrology department due to a 3-week deterioration in general health, marked by fever up to 39 °C and diarrhea. His medical background included chronic active antibody-mediated (cABMR) rejection for the past 2 years with donor-specific antibody (DSA) below the positivity threshold, hypertension, mellitus diabetes managed with vildagliptin 50 mg/day, and a history of squamous cell carcinoma. His immunosuppressive regimen consisted of tacrolimus, mycophenolate mofetil, prednisone, and monthly photopheresis session. At baseline, his serum creatinine was 100 μmol/L with a urine protein-to-creatinine ratio (UPCR) of 0.5 g/g.

**FIGURE 1:**
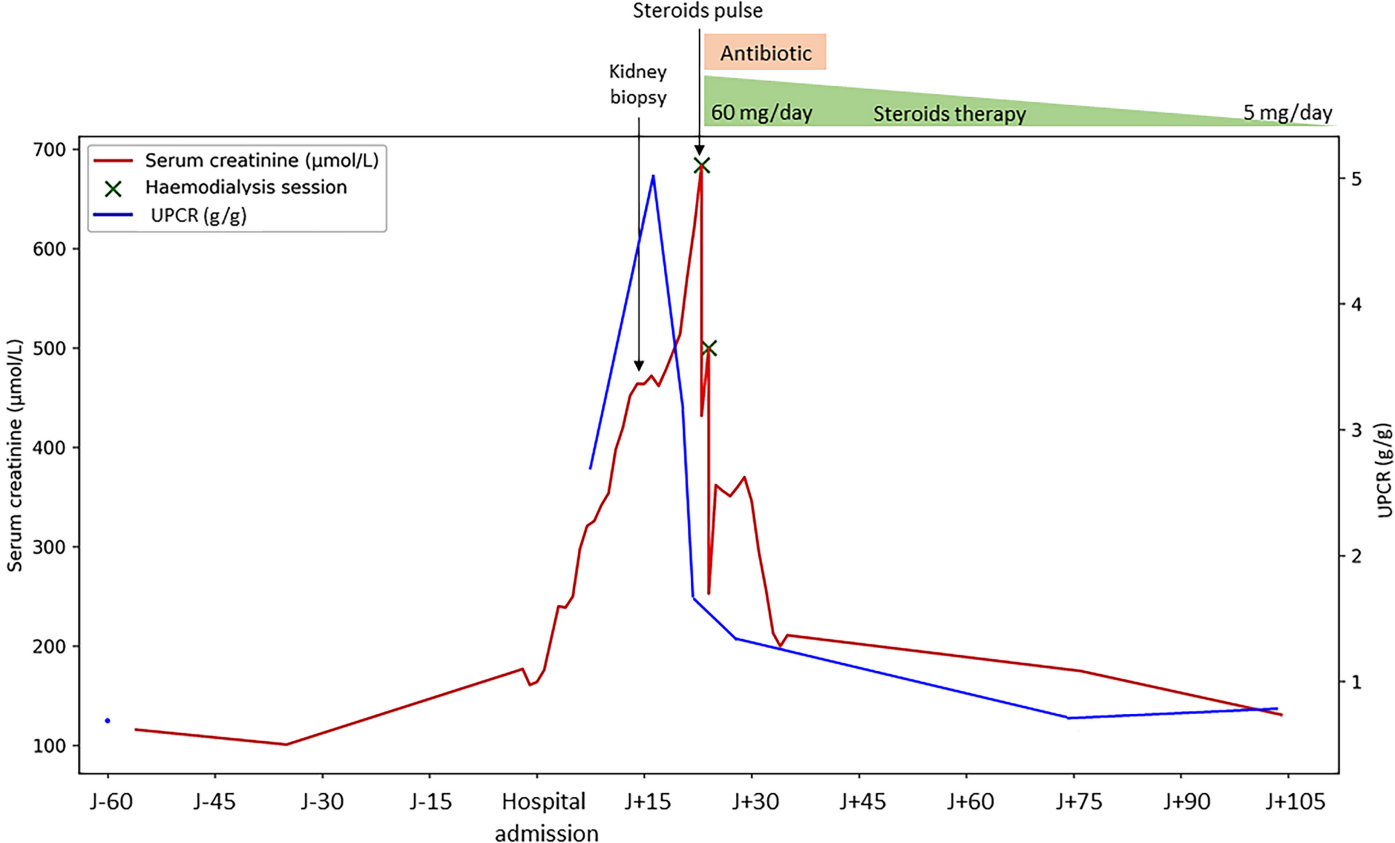
Clinical course of the patient during hospitalization (J0, hospital admission; UPCR, urine protein creatinine ratio).

At admission on examination, the patient was febrile but hemodynamically stable, with no lymphadenopathy or skin lesions or peripheral edema. Renal function had deteriorated significantly: serum creatinine was 180 μmol/L, and UPCR had increased to 2.7 g/g. During hospitalization, his condition worsened, with serum creatinine peaking at 684 μmol/L and the onset of nephritic syndrome (UPCR 5.1 g/g, albumin 22 g/L with hypertension and peripheral edema (+6 kg over 1 week), accompanied by oliguria. Ultrasound of the kidney graft showed no structural abnormalities. Urinalysis revealed microscopic hematuria (3455 RBC/mm^3^) and pyuria (279 WBC/mm^3^). The urinary Na/K ratio was >1, consistent with acute organic renal failure. Given the severe renal impairment and oliguria, the patient required hemodialysis due to the absence of renal function recovery and fluid overload. The patient’s clinical course was illustrated in Figure 1.

**FIGURE 2:**
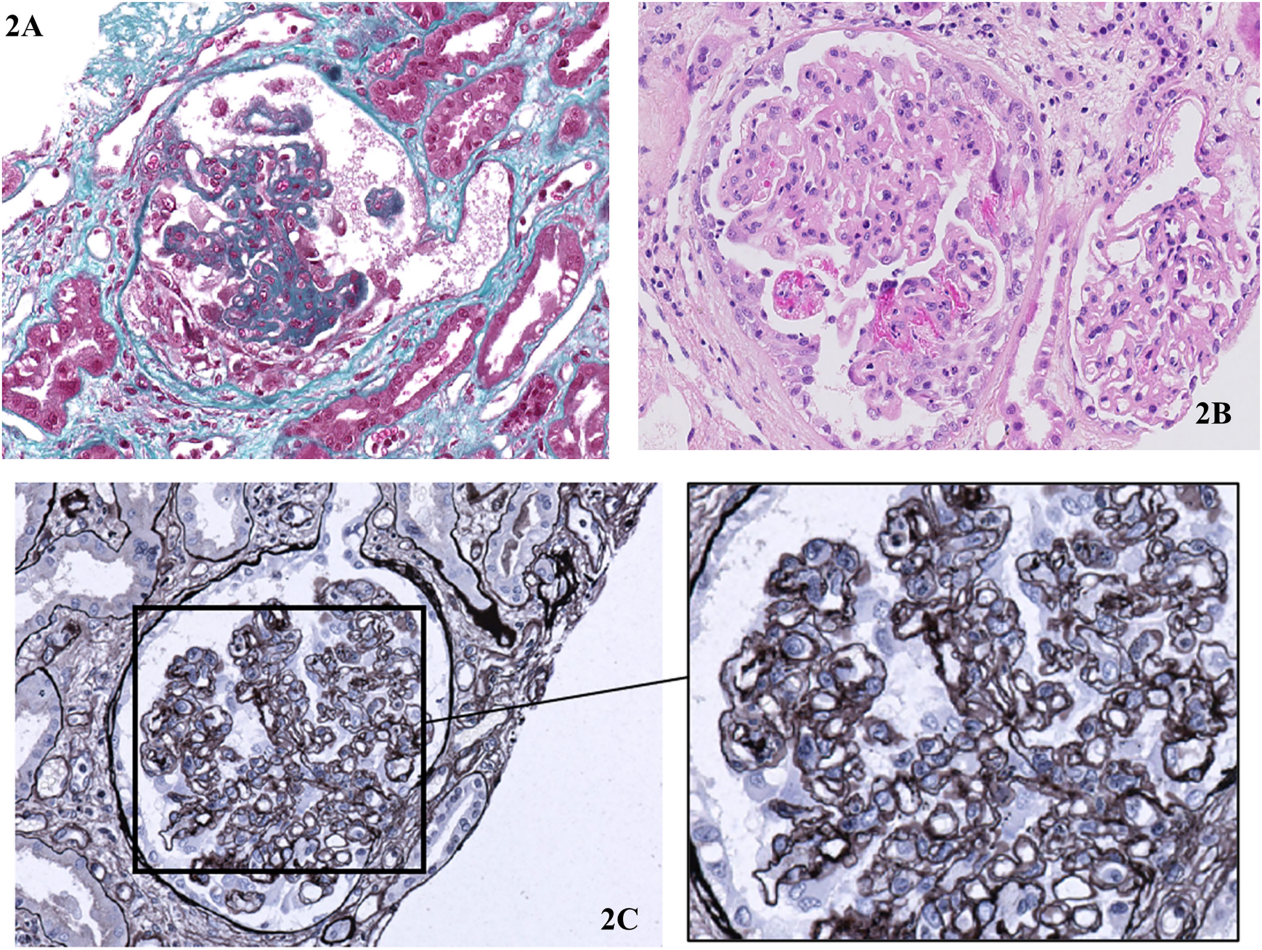
**(A)** Masson trichrome stain showing active cellular extracapillary glomerulonephritis with fibrous crescents in one-third of glomeruli. Magnification × 200. **(B)** Hematoxylin and eosin stain highlighting necrotizing lesions and cellular proliferation within glomeruli. Magnification × 200. **(C)** Jones stain demonstrating diffuse duplication of the glomerular basement membrane, consistent with chronic antibody-mediated rejection. Magnification × 2,400.

A renal biopsy was performed (Figure 2) and revealed active cellular extracapillary glomerulonephritis in one-third of glomeruli, with 25% globally sclerotic glomeruli, interstitial fibrosis, and 20% tubular atrophy. Capillaritis and glomerulitis were present, consistent with chronic antibody-mediated rejection, along with diffuse glomerular basement membrane duplication. The Banff 2022 score was g3, cg3, i0, iFIAT1, t0, ti1, ci1, ct1, v0, cv1, ah3, cpt2, C4d0. Immunofluorescence revealed irregular granular staining for IgM, IgA, and C3, with focal C1q deposition, but no IgG deposits or peritubular C4d labeling.

Immunological evaluation revealed a low CH50 (14.8 U/mL normal range 41.7–95.1), low C3 (0.275 g/L normal range 0.81–1.57 UI/mL), and normal C4 (0.190 g/L normal range 0.129–0.392 UI/mL). Rheumatoid factor was positive (12.9 IU/L), and plasma protein electrophoresis with immunofixation revealed two non-quantifiable monoclonal IgA lambda and IgG kappa, consistent with type III cryoglobulinemia (polyclonal IgG 40 mg/L, IgM 83 mg/L). ANCA and anti-glomerular basement membrane antibodies were negative. Infectious workup, including stool cultures, blood cultures, and cytomegalovirus PCR, returned negative results. Two transthoracic echocardiograms performed one week apart and PET scan ruled out infective endocarditis.

Reinterrogation after renal biopsy results revealed a cat bite 2 months prior, empirically treated by his primary care physician with amoxicillin-clavulanate. This prompted *Bartonella henselae* serology on initial serum, which was positive (IgM > 384, IgG 256), and seroconversion confirmed 15 days later (IgM > 384, IgG 1024), establishing the diagnosis of infectious crescentic glomerulonephritis associated with *Bartonella henselae* 15 days after the hospital admission. The *Bartonella henselae* PCR in whole blood was negative.

### Treatment and evolution

The patient was treated with azithromycin (500 mg/day for 5 days) to target the *Bartonella henselae* infection and a corticosteroid regimen (5 mg/kg bolus followed by 20 mg/day for 15 days, tapered by 5 mg every 2 weeks to a maintenance dose of 5 mg/day) to manage the CrGN. Renal function improved progressively, allowing discontinuation of hemodialysis after two sessions. Serum creatinine decreased from a peak of 684–211 μmol/L within the first month of treatment. However, the patient developed high-grade gastrointestinal bleeding, likely related to corticosteroid use, requiring endoscopic clipping (5 clips) and transfusion of 7 units of red blood cells. The patient’s general condition significantly improved, with resolution of fever and normalization of inflammatory markers (CRP 8 mg/L), CH50 levels normalized, and plasma protein electrophoresis with immunofixation no longer detected monoclonal gammopathies (IgG and IgA). Nephroprotective therapy was optimized: irbesartan, which was already prescribed at baseline (75 mg/day), was up titrated to 150 mg/day, and dapagliflozin (10 mg/day) was newly introduced to further reduce proteinuria and preserve renal function.

At 1-month follow-up, serum creatinine stabilized at 250 μmol/L, and proteinuria decreased to 2.8 g/g. By 3 months, renal function continued to improve, with serum creatinine at 210 μmol/L and proteinuria at 1.5 g/g. *Bartonella henselae* serology showed a decline in antibody titers (IgM < 48, IgG 128), confirming effective treatment of the infection. At 6-month follow-up, serum creatinine was 130 μmol/L, and proteinuria had decreased to 1.2 g/g. The patient remained clinically stable, with no signs of active infection or rejection. At 1-year follow-up, renal function was preserved with a serum creatinine of 120 μmol/L and UPCR of 0.5 g/g, returning to baseline levels. Immunosuppressive therapy was maintained at stable doses.

The final diagnosis was *Bartonella henselae-*associated post infectious CrGN, occurring without endocarditis, in a KTx recipient, successfully managed with targeted antibiotic therapy and corticosteroids.

## Discussion

This case highlights a rare presentation of crescentic glomerulonephritis associated with *Bartonella henselae* infection in a KTx recipient, diagnosed without evidence of infective endocarditis. Unlike typical cases where *Bartonella*-related renal damage occurs in the context of endocarditis or systemic vasculitis, this patient’s diagnosis was uncovered through targeted re-interrogation after renal biopsy results revealed immune complex-mediated glomerulonephritis. The absence of classic symptoms (e.g., lymphadenopathy, angiomatosis) initially obscured the diagnosis, emphasizing the critical role of a thorough clinical history—particularly in immunocompromised patients—when faced with unexplained glomerulonephritis.

The diagnostic breakthrough occurred when the patient, upon re-questioning, reported a cat bite 2 months prior, treated empirically with amoxicillin-clavulanate. This detail, initially overlooked, became pivotal after biopsy findings (IgM/IgA/C3 deposits, complement consumption) suggested an infectious or immune-mediated trigger. Also, the finding of CrGN with complement consumption is frequently observed in *Bartonella*-associated glomerulonephritis, distinguishing it clinicopathologically from other bacterial endocarditis-related glomerulonephritis (14). Seroconversion for *Bartonella henselae* (IgM > 384, IgG rising from 256 to 1,024) confirmed the diagnosis, underscoring that repeated patient interrogation, guided by histopathological clues, is essential in atypical presentations of CrGN. The negative *Bartonella* PCR in this case does not exclude the diagnosis, as PCR sensitivity for detecting *Bartonella. henselae* is often low, and serology remains the gold standard for diagnosis (13). According to the updated 2023 Duke criteria (15), *Bartonella* serology (IgM > 384, IgG 1024) does not meet major criteria for infective endocarditis (IgG titer lesser than 1800), while fever and immune complex-mediated CrGN fulfilled minor criteria. However, no vegetations or valvular abnormalities or secondary localization were detected—despite thorough investigation including two transthoracic echocardiograms and PET scan—excluding a definitive diagnosis of infective endocarditis. As highlighted by Schaack et al. (13), *Bartonella* can cause isolated glomerulonephritis without clinically overt infective endocarditis. Moreover, the favorable outcome without endocarditis-specific antibiotic therapy suggests that it was well an isolated *Bartonella*-associated CrGN in this patient.

The pathophysiological mechanism in this case likely involves immune complex deposition, as evidenced by granular IgM/IgA/C3 staining and low complement levels. The atypical absence of IgG deposits despite the presence of IgM, IgA, and C3 is a recognized pattern in *Bartonella*-related glomerulonephritis (see Table 1). This may reflect preferential activation of the alternative complement pathway and a blunted IgG class-switch response in transplant recipients on long-term immunosuppression (11–13). The low CH50 with preserved C4 observed in this patient further supports predominant alternative pathway activation rather than classical pathway engagement. Regarding the type III cryoglobulinaemia, several arguments strongly favor an infectious etiology rather than a rejection-driven process. Type III cryoglobulinaemia is well-recognized as a complication of bacterial infections, including *Bartonella*, and is far less commonly associated with antibody-mediated rejection. Furthermore, complement levels had been normal prior to this episode, including during the known period of cABMR activity, arguing against rejection-driven complement consumption. The cABMR was moreover well controlled at the time of presentation, with stable renal parameters and negative DSA. Taken together, the complete resolution of cryoglobulinaemia following antibiotic and corticosteroid treatment provides the strongest argument in favor of a primarily infectious etiology.

**TABLE 1:** Comparison of reported cases of isolated *Bartonella*-associated glomerulonephritis without infective endocarditis in renal transplant recipients.

References	Patient	Histology	Serology	Endocarditis	Treatment	Outcome
Chaudhry et al. (11)	KTx recipient, adult	Pauci-immune CrGN, p-ANCA	IgG^+^ IgM^+^	No	Doxycycline+ ↘ immunosuppression	Allograft loss
Pischel et al. (12)	KTx recipients	MPGN	IgG^+^ IgM^+^	No	Azithromycin + doxycycline	Dialysis, then recovery
Schaack et al. (13)	KTx recipient, adult	CrGN, IgM/IgA/C3, no IgG	IgM^+^ IgG^+^	No	Azithromycin	Dialysis, then partial recovery
Present case, 2026	KTx recipient, adult	CrGN, IgM/IgA/C3, no IgG, type III cryo	IgM > 384, IgG > 1,024	No	Azithromycin + steroids	Dialysis, then full recovery (Cr 120 μmol/L at 1 year)

KTx, kidney transplant; CrGN, crescentic glomerulonephritis; MPGN, membranoproliferative glomerulonephritis; Cr, serum creatinine.

Therapeutically, the combination of azithromycin (for *Bartonella*) and corticosteroids (for CrGN) led to rapid renal recovery, with serum creatinine improving from 684 to 211 μmol/L. The successful therapeutic outcome in our case contrasts sharply with the outcome reported by Chaudhry et al. (11) where glomerulonephritis progressed to end-stage renal disease and allograft loss. It is possible, as these authors suggest, that a delay in initiating effective therapy may have contributed to the development of post-infectious complications leading to graft failure. This reinforces the key message of our case: rapid consideration of *Bartonella* as an etiology and prompt initiation of adequate treatment are paramount for the preservation of allograft function. The later addition of irbesartan and dapagliflozin further reduced proteinuria (UPCR from 5.1 to 0.5 g/g at 1 year), highlighting the importance of nephroprotective strategies in preserving long-term graft function. The occurrence of gastrointestinal bleeding under corticosteroids serves as a reminder of the delicate balance between immunosuppression and anti-inflammatory therapy in transplant recipients. Prophylactic proton pump inhibitor therapy should be routinely co-prescribed in transplant patients receiving high-dose corticosteroids, particularly those with additional risk factors such as prior gastrointestinal history or anticoagulation. Importantly, immunosuppression was not modified, and no new rejection episodes occurred during follow-up, consistent with findings from propensity score-matched analyses showing that stable immunosuppression is feasible in post-transplant infectious CrGN if the infection is effectively treated (16).

This case underscores three clinical lessons for clinical practice: (i) Re-interrogation is critical: In CrGN of unclear etiology, a second, targeted interview—focusing on exposure risks (e.g., animal bites, travel, occupational hazards)—should be systematically performed after biopsy results. Infectious triggers like *Bartonella* may lack classic symptoms in immunocompromised hosts. (ii) Biopsy guides diagnosis: Histopathological findings (e.g., immune deposits, complement activation) should prompt a re-evaluation of clinical history and targeted serological testing, even for rare pathogens. (iii) Multidisciplinary management: Collaboration between nephrologists, infectious disease specialists, and pathologists is essential to navigate diagnostic challenges and optimize therapy in complex cases. (iv) Corticosteroid safety in transplant recipients: High-dose steroid therapy warrants routine proton pump inhibitor prophylaxis to mitigate the risk of upper gastrointestinal bleeding. Future research should explore the mechanism of atypical post infectious triggers in CrGN in transplant recipients.

## Data Availability

The raw data supporting the conclusions of this article will be made available by the authors, without undue reservation.
